# The impact of glutamine supplementation on the symptoms of ataxia-telangiectasia: a preclinical assessment

**DOI:** 10.1186/s13024-016-0127-y

**Published:** 2016-08-18

**Authors:** Jianmin Chen, Yanping Chen, Graham Vail, Heiman Chow, Yang Zhang, Lauren Louie, Jiali Li, Ronald P. Hart, Mark R. Plummer, Karl Herrup

**Affiliations:** 1 Department of Cell Biology and Neuroscience, Rutgers University, 604 Allison Road, Piscataway, NJ 08854 USA; 2 Division of Life Science, The Hong Kong University of Science and Technology, Clear Water Bay, Kowloon, Hong Kong; 3 Kunming Institute of Zoology, Chinese Academy of Sciences, Kunming, Yunnan, China

**Keywords:** Glutamine, Ataxia-telangiectasia, Alzheimer’s disease, ATM

## Abstract

**Background:**

Our previous studies of Alzheimer’s disease (AD) suggested that glutamine broadly improves cellular readiness to respond to stress and acts as a neuroprotectant both in vitro and in AD mouse models. We now expand our studies to a second neurodegenerative disease, ataxia-telangiectasia (A-T). Unlike AD, where clinically significant cognitive decline does not typically occur before age 65, A-T symptoms appear in early childhood and are caused exclusively by mutations in the ATM (A-T mutated) gene.

**Results:**

Genetically ATM-deficient mice and wild type littermates were maintained with or without 4 % glutamine in their drinking water for several weeks. In ATM mutants, glutamine supplementation restored serum glutamine and glucose levels and reduced body weight loss. Lost neurophysiological function assessed through the magnitude of hippocampal long term potentiation was significantly restored. Glutamine supplemented mice also showed reduced thymus pathology and, remarkably, a full one-third extension of lifespan. In vitro assays revealed that ATM-deficient cells are more sensitive to glutamine deprivation, while supra-molar glutamine (8 mM) partially rescued the reduction of BDNF expression and HDAC4 nuclear translocation of genetically mutant Atm^−/−^ neurons. Analysis of microarray data suggested that glutamine metabolism is significantly altered in human A-T brains as well.

**Conclusion:**

Glutamine is a powerful part of an organism’s internal environment. Changes in its concentrations can have a huge impact on the function of all organ systems, especially the brain. Glutamine supplementation thus bears consideration as a therapeutic strategy for the treatment of human A-T and perhaps other neurodegenerative diseases.

## Background

Glutamine (Gln or Q) is the most abundant free amino acid in the human blood stream. Normally, the body can make enough glutamine for its needs and under these conditions glutamine is a non-essential amino acid. Yet in times of stress glutamine is depleted from the blood stream faster than it can be produced in muscle and other tissues. Under these conditions, cells become dependent on an exogenous supply of glutamine. This context-dependent shift has led to the classification of glutamine as a “conditionally essential” amino acid. In the central nervous system, brain glutamine is the major substrate for the generation of both excitatory and inhibitory neurotransmitters (glutamate and γ-aminobutyric acid). It is also a vital source of energy for the nervous system as it feeds directly into the tricarboxylic acid (TCA) cycle, the main source of ATP in the cell.

We have previously shown the neuroprotective value of glutamine supplementation in vitro and in vivo [1]. Specifically, short term oral glutamine supplementation significantly reduces the biochemical indices of neurodegeneration in mouse models of Alzheimer’s disease. The benefits of glutamine supplementation were broad spectrum and included reduction of tau phosphorylation, blockage of neuronal cell cycle reentry, improvement in the DNA damage response and a reduction in synaptic protein loss. As part of this earlier study we showed that low glutamine decreases the abundance of several key stress response proteins, including ATM (ataxia-telangiectasia mutated).

ATM deficiency, caused by mutations in the ATM gene, underlies a childhood neurodegenerative disease known as ataxia telangiectasia (A-T). Due to the loss of cerebellar Purkinje and granule cells, patients with A-T suffer from uncoordinated or ataxic movements beginning at 2–3 years of age [2]. A-T patients also have increased mortality because of cancer, respiratory system infections, and various other rare complications. The median life-span of an individual with A-T is about 23 years [3]. Affected individuals can also develop cardiovascular disease, accelerated aging, and insulin resistance. Systemic inflammation may contribute to these disease phenotypes as an immune challenge significantly alters the timing and severity of the A-T phenotype in *Atm*^*−/−*^ mouse brain [4, 5]. Although there are no reports of an inflammatory reaction in their brains, A-T patients have elevated serum IL8, a pro-inflammatory CXC chemokine and neutrophil chemoattractant associated with premature cellular senescence and chronic inflammatory disorders [6]. Notably, recent studies have suggested that glutamine plays an important role in regulating IL8 secretion; deprivation of glutamine stimulates IL8 secretion in U2OS osteosarcoma cells and A549 lung cancer cells as well as in human A-T fibroblasts [7, 8].

Beyond these immune system abnormalities, A-T children are known to have a lower body mass index and often fail to thrive [9–11]. A recent study by Ross et al. demonstrated profound malnutrition in A-T patients and the need for early nutritional intervention [12]. These observations plus the neuroprotective effect seen in mouse models of Alzheimer’s disease led us to hypothesize that glutamine might be beneficial in A-T as well as in AD. In this study, we present multiple lines of evidence from *Atm*^*−/−*^ mice in support of this hypothesis. We suggest that glutamine supplementation has significant potential as part of a therapeutic regimen for the treatment of A-T.

## Methods

### A-T mouse models

For our A-T model, we chose the *Atm*^*tm1Awb*^ mutant allele [13] and the *Atm*^*tm1Bal*^ mutant allele [14] from The Jackson Laboratories. All in vivo experiments were done using *Atm*^*tm1Awb*^ strand. For primary neuron culture, both strands were used. All animal procedures were performed according to Rutgers University Institutional Animal Care and Use Committee standards.

### Glutamine supplement

For glutamine supplementation experiments, 4 % glutamine (Sigma) in sterile tap water was made fresh daily and offered as the sole source of drinking water for 5 consecutive days (Monday through Friday). Control mice were fed with only sterile tap water. To avoid undue stress from elevated ammonia concentrations, all mice drank glutamine-free water 2 days each week (Saturday and Sunday).

### Blood glucose and glutamine measurements

About 0.3 ml blood was collected by quick sub-mandibular bleeding from mice before sacrifice. Non-fasting blood glucose concentration was measured using the Roche Accu-Chek Aviva Plus Blood Glucose Meter. For glutamine assays, blood samples were deproteinized immediately using the Deproteinizing Sample Preparation Kit from BioVision following the manufacture’s protocol. Deproteinized samples were kept in the −80° freezer before assay. Blood glutamine concentration was measured using EnzyChrom™ Glutamine Assay Kit from BioAssay Systems following the manufacturer’s protocol.

### Primary neuronal cell cultures

Timed pregnancies were established from wild type ICR mice (Taconic Biosciences) or wild type mice from *Atm*^*−/−*^ colonies. Embryos were harvested on embryonic day 15.5–16.5 for cortical neuron culture as previously described [15]. Neurons were cultured in normal medium with 2 mM of a glutamine-alanine dipeptide (GIBCO® GlutaMAX™, Life Technologies) as a source of glutamine. Culture media were changed every 4–5 days. On DIV9–11, neurons were fed with fresh medium with different concentrations of Glutamax. 72 h later, on DIV12–14, neurons were collected for immunostaining or western blotting or gene expression analysis. To block endogenous glutamine production, neurons were treated with a combination of 5 mM glutamine synthase (GS) inhibitor methionine sulfoximine (MSO) (Sigma). Either ATM-specific inhibitor Ku55933 (10 μM) or Ku60019 (2 μM) (Calbiochem, currently Merck Millipore) was used to inhibit ATM activity in wild type neurons. shRNA against Atm was used to study the effect of gene knockdown. shRNA against mouse ATM [pGFP-C-shLenti-ATM (TL500154), OriGene] were transfected with Lipofectamine LTX (Life Technologies). Six hours after transfection, cells were refreshed with culture medium and further incubated for 48 h for gene knockdown prior to being challenged at different glutamine concentrations for another 72 h.

### Western blots and immunocytochemistry

For western blot analysis, cultured neurons were lysed in RIPA buffer (Thermo Scientific) containing proteinase and phosphatase inhibitors (Roche Diagnostics). Equal amounts of protein were separated on 4–20 % SDS PAGE gradient gels (Bio-Rad) and then transferred to PVDF or nitrocellulose membranes (Bio-Rad) for immunodetection. Primary antibodies used were: ATM2c1 and 53BP1 (Abcam); Actin (Santa Cruz Biotechnology); GFAP, GS, mTOR and P-mTORs2448 (Cell Signaling); and tau3R (Millipore). Secondary antibodies were chicken anti-rabbit IgG-HRP and chicken anti-mouse IgG-HRP (Santa Cruz Biotechnology). Chemiluminescent substrates used were SuperSignal™ West Pico Chemiluminescent Substrate and SuperSignal™ West Femto Maximum Sensitivity Substrate (Thermo Scientific). For immunofluorescence staining, neurons were fixed in 4 % paraformaldehyde for 30 min. Fixed cells were then incubated in blocking buffer (10 % goat serum, 0.5 % Triton X100 in PBS) for 1 h. Primary antibody HDAC4 (1:1000, Abcam) incubation were carried out in 4°, overnight. Alexa linked secondary antibodies (Life Technologies) were used to detect the presence of the HDAC4 antigens. Stained cells were photographed and viewed at a final magnification of 200 using Leica Application Suite/Leica DM5000B.

### RT PCR and real-time PCR

Total RNA was purified using TRIzol reagent (Life Technologies) following the standard protocol. For real-time PCR, cDNAs were generated from total RNA using High Capacity cDNA Reverse Transcription Kit (ABI). The PCR reactions were done using iTaq Universal SYBR Green Supermix (Bio-RAD) on an ABI PRISM® 7900HT Sequence Detection System. Primer sets used for real-time PCR include:

- For 36B4 (control)
- Forward: atcgtctttaaaccccgcgt
- Reverse: acgttgtctgctcccacaat;
- For mBDNF (common region)
- Forward: gaaggctgcaggggcatagacaaa
- Reverse: tacacaggaagtgtctatccttatg.

Because PCR products for BDNF exons 2, 3 and 4 were larger than optimal for real-time PCR analysis, expressions of these exons were analyzed by RT-PCR using the SuperScript® III One-Step RT-PCR System with Platinum® Taq DNA Polymerase kit (Life Technologies) on a Techne PrimeG gradient enabled thermal cycler. PCR products were resolved in 1.5 % agarose gel and visualized by staining with ethidium bromide. Relative expression concentrations were determined using ImageJ from NIH. Primer sets used for RT-PCR were:

- BDNF exon2
- Forward: ggaagtggaagaaaccgtctagagca
- Reverse: gaagtgtacaagtccgcgtcctta
- BDNF exon3
- Forward: gctttctatcatccctccccgagagt
- Reverse: gaagtgtacaagtccgcgtcctta
- BDNF exon4
- Forward: ctctgcctagatcaaatggagcttc
- Reverse: gaagtgtacaagtccgcgtcctta
- 36B4
- Forward: atcgtctttaaaccccgcgt
- Reverse: acgttgtctgctcccacaat

### Field potential recording

Extracellular recordings of field EPSPs (fEPSPs) were made with ACSF-filled glass electrodes (5–10 μm tip diameter) according to protocols previously established in our lab [16]. For baseline recordings, test stimuli (0.1 ms) were delivered with a bipolar platinum/iridium stimulating electrode at 1 min intervals. For recordings of CA1 activation by Schaffer collateral stimulation, recording and stimulating electrodes were both placed in stratum radiatum. Each experiment was begun by obtaining input–output relationships to establish the strength of baseline synaptic transmission. A Grass S8800 stimulator connected to a Grass PSIU6 photoelectric stimulus isolation unit was used to deliver a series of increasing intensity constant current pulses. Current magnitude is adjusted to elicit responses ranging from just-suprathreshold to near maximal. Following this, stimulus intensity was adjusted to evoke fEPSPs 30–40 % of maximum, typically 30–40 μA. To elicit long-term potentiation (LTP), theta burst stimulation (TBS) was used. A single stimulus consists of 6–12 bursts of four 100 Hz pulses spaced 200 ms apart. Response magnitude was quantified using the slope of the field potential.

### Microarray data

Human A-T and mouse *Atm*^*−/−*^ gene expression datasets were published previously [17] and are available from the NIH GEO archive under accession numbers GSE50951 and GSE61019.

### Statistics

For lifespan study, the significance of the difference between the glutamine and control curves was determined by the Mantel-Cox log-rank test. For the rest of the studies, the p values were determined by Student *t*-test. *** denotes *p* < 0.001; ** denotes *p* < 0.01; * denotes *p* < 0.05; n.s. means *p* ≥ 0.05.

## Results

### Glutamine modulates the metabolomics of ATM deficiency

**Fig. 1:**
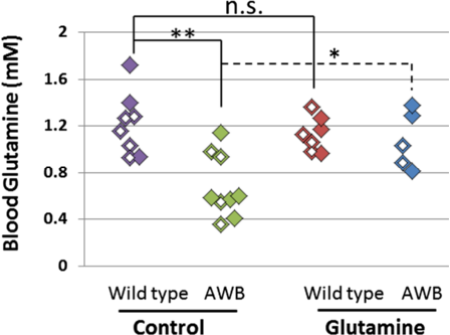
Oral glutamine supplementation increases non-fasting blood glutamine concentrations in *Atm* ^*−/−*^ mice. *Atm* ^*−/−*^ mice (*green or blue symbols*) have significantly lower glutamine blood glutamine compared to age-matched wild type mice (*purple or red symbols*). Glutamine supplementation significantly improves the blood glutamine deficit of *Atm* ^*−/−*^ mice but has no effect on wild type mice. Symbols with a white square inside indicate female mice. Statistically different by two-tailed *t*-test: **, *p* < 0.01; *, *p* < 0.05; n.s. (not significant) *p* ≥ 0.05

Our previous research has shown that glutamine has a broad neuroprotective effect [1], so we became interested in whether glutamine deficiency contributed to the A-T phenotype. As such, we first asked whether *Atm*^*tm1Awb/tm1Awb*^ mice (referred to as *Atm*^*−/−*^ or AWB) presented with any evidence of glutamine deficiency. Blood glutamine concentrations of *Atm*^*−/−*^ mice were measured (see Methods) and found to be 25 % below those in age-matched control mice (Fig. 1, *p* < 0.001). Feeding glutamine for 2-weeks did not change the glutamine concentration in wild type mice (*p* = 0.5); however, glutamine supplementation significantly increased the concentration of blood glutamine in *Atm*^*−/−*^ mice (*p* < 0.02), raising it to levels comparable to wild type. These observations suggest first that blood glutamine concentration is tightly regulated in the wild type as extra glutamine has little effect and second that the *Atm*^*−/−*^ mouse is intrinsically glutamine deficient, thus explaining why glutamine supplementation helps to restore normal serum levels.

**Fig. 2:**
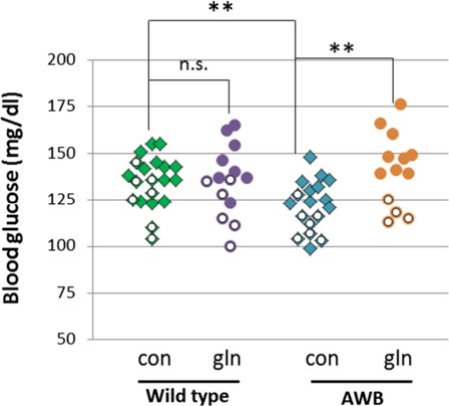
*Atm* ^*−/−*^ mice have significantly lower blood glucose concentration compared to their wild type littermate mice. Glutamine supplementation of the *Atm* ^*−/−*^ mice significantly increased their blood glucose concentration. Unlike *Atm* ^*−/−*^ mice however, feeding glutamine had no effect on blood glucose of wild type mice. This effect was sexually dimorphic with a strong effect in males, but no effect observed in female *Atm* ^*−/−*^ mice. Symbols with a white circle inside indicate female mice. Statistically different by two-tailed *t*-test: **, *p* < 0.01; n.s. (not significant) *p* ≥ 0.05

Glutamine is believed to be important for maintaining euglycemia by modulating secretion of glucagon-like peptide 1 (GLP-1) [18–20]. As ATM deficiency is already associated with defective glucose homeostasis and mild diabetes [21, 22], we predicted that blood glucose concentrations would also be reduced in A-T. Thus, we first measured the non-fasting blood glucose concentrations of our glutamine treated and untreated animals (Fig. 2). In wild type mice from our *Atm* colony, resting blood glucose concentrations were 136 ± 11 mg/dl. ATM-deficient littermates had only 122 ± 14 mg/dl blood glucose, a significant reduction (*p* < 0.01). Thus, as in humans, ATM deficiency impairs glucose metabolism in ATM-deficient mice. We next asked whether oral glutamine supplementation might have an impact on this metabolic imbalance. In wild-type animals, oral glutamine supplementation had no effect on blood glucose concentrations (*p* = 0.99). In contrast, in *Atm*^*−/−*^ mice, an 8-week regimen of oral glutamine supplementation increased blood glucose significantly (*p* < 0.05), restoring the values to near wild type levels. Of note, this increase was observed almost exclusively in the male mice; there was little difference in the glucose levels of female mice. The cause for this gender difference has not been ascertained. However, it is still clear that glutamine supplementation can restore proper blood glucose levels when they are abnormal, yet has no effect on the homeostatic mechanisms of the wild type animals, a finding of clinical significance.

**Fig. 3:**
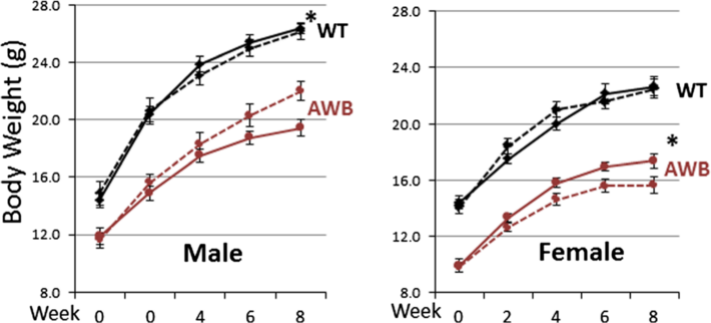
Glutamine supplementation improves the body weight of *Atm* ^*−/−*^ mice in a gender-specific manner. Male *Atm* ^*−/−*^ mice gained weight significantly faster during their 8-week supplementation with glutamine. The rate of weight gain in glutamine-fed female *Atm* ^*−/−*^ was significantly slower than female *Atm* ^*−/−*^ mice on regular drinking water. Symbols: solid lines = control drinking water; dotted lines = glutamine supplementation. There were 8–10 mice in each of the 8 study groups, and a total of 69 mice were used in the body weight study. Statistically different by two-tailed *t*-test: *, *p* < 0.05. Error bars = standard errors

Consistent with the metabolic changes observed, we found that the total body weight of the mice was also affected by the addition of glutamine to their diet. Once again, the supplement had no effect on the wild-type controls; their developmental weight gain was identical with and without supplementation. Consistent with previous findings, *Atm*^*−/−*^ mice of both sexes weigh significantly less than their wild-type counterparts at all ages. Further, in these ATM deficient mice, glutamine supplementation had a significant effect on their size. Intriguingly, this effect also showed a strong sexual dimorphism. In male mice, the supplement enabled the animals to gain weight much faster than their un-supplemented littermates (although even 2 months of supplementation were not enough to allow them to catch up with the wild-type animals). In female *Atm*^*−/−*^ mice, however, the exact reverse was observed: glutamine supplementation retarded their developmental weight gain (Fig. 3). This male/female difference in *Atm*^*−/−*^ mice is not seen in un-supplemented animals and has no known precedent in the A-T clinical literature.

### Glutamine supplementation modulates synaptic plasticity

**Fig. 4:**
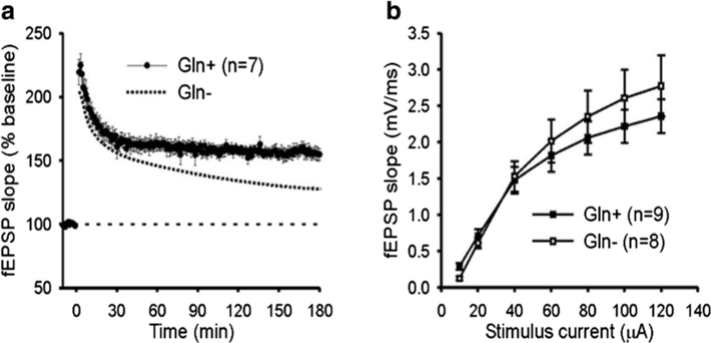
LTP in *Atm−/−* animals can be improved by dietary supplementation with glutamine. **a**. Plots of fEPSP slope, normalized to baseline, showing the responses of *Atm−/−* animals to the 6xTBS induction protocol. After 10 min of baseline recording, the TBS train was delivered at time = 0. Filled symbols show the average response of animals that received glutamine. Dotted line shows the average response of untreated *Atm*−/− animals from a separate parallel study [23]. **b**. Input–output plot of fEPSP slope in response to single pulses of increasing amplitude. Stimulus intensities of 10, 20, 40, 60, 80, 100, and 120 μA were tested. There was not a significant difference between *Atm−/−* animals with and without glutamine (*p* > 0.3, *t*-test, two-sided). Error bars = standard deviations

We have shown previously [16] that *Atm*^*−/−*^ mice have a significant deficit in TBS-induced Schaffer collateral LTP (long term potentiation) as compared to wild-type mice. To see if glutamine supplementation would be efficacious in improving this deficit, we evaluated LTP magnitude in homozygous *Atm*^*−/−*^ animals with glutamine treatment. Hippocampal synaptic activity was assessed with field potential recording of the CA3-CA1 synaptic pathway. To avoid ceiling effects, LTP was evoked with trains containing 6 theta bursts (6xTBS), which allows both increased and decreased response magnitude to be detected. A cohort of *Atm*^*−/−*^ animals that received glutamine supplementation in their diet showed elevated LTP (153 ± 4.7 % above baseline; Fig. 4a, filled symbols) as compared to untreated animals from a separate parallel study (127 ± 8.1 % above baseline; Fig. 4a, dotted line) [23]. The improvement in synaptic strength was limited to late LTP; the early increases in synaptic strength that decayed within the first 30 min post-6xTBS did not differ between treated and untreated animals. To assess baseline synaptic strength, we also obtained input–output relationships for *Atm*^*−/−*^ mice with and without glutamine supplementation (Fig. 4b). The relationship between stimulus current and slope of the fEPSP was comparable to wild-type animals and did not differ significantly between the two groups of animals (*p* > 0.3). This suggests that restoration of LTP by glutamine does not result from an overall increase in synaptic strength but rather is limited to an effect on synaptic plasticity.

### Glutamine prolongs the lifespan of the ATM^−/−^ mice

**Fig. 5:**
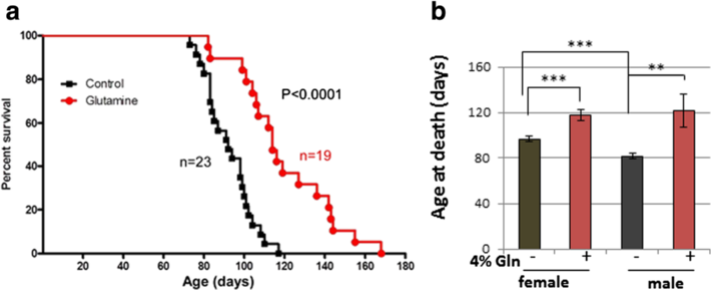
Glutamine supplementation significantly increases the life span of *Atm* ^*−/−*^ mice. **a** Kaplan-Meyer curves of the mice. *Atm* ^*−/−*^ mice on normal diet (control, squares) had a mean lifespan of 85 days (16 females and 7 males); *Atm* ^*−/−*^ mice with glutamine supplementation (circles) had a mean lifespan of 120 days (13 females and 6 males). The significance of the difference between the glutamine and control curves was determined by the Mantel-Cox log-rank test. **b** Glutamine significantly extends life span of both male and female *Atm* ^*−/−*^ mice. The mean age at death is plotted separately for male (M) and female (F) *Atm* ^−/−^ mice either with (Gln) or without (Cont) dietary glutamine supplementation. Statistically different by two-tailed *t*-test: ***, *p* < 0.001; **, *p* < 0.01. Values represent means and standard errors

The observations of improvement in hippocampal circuitry (synaptic plasticity), blood glucose, blood glutamine, and body weight gain led us to ask whether these individual improvements following glutamine supplementation might work together in such a way that there was an overall impact on the life-span of the mutant mice. To address this question, we randomly divided a cohort of 42 *Atm*^*−/−*^ mice into two groups. One group was fed drinking water supplemented with 4 % glutamine; the other group had only normal water to drink. Normally, in our colony, the average life span of an *Atm*^*−/−*^ mutant mouse is approximately 85 days (Fig. 5a black symbols). Glutamine supplementation led to a significant increase in this figure. We began glutamine treatment at 4–5 weeks of age, and found that this supplementation increased the lifespan of ATM-deficient mice by nearly one third to 120 days (*p* < 0.0001; Fig. 5a, red symbols). Given that in female mice glutamine has no effect on blood glucose concentration and even leads to lower weight gain, we were particularly intrigued that this effect on life span was seen in both males and females. Nonetheless, as the mutant males in our colony have significantly shorter lifespans than the mutant females (*p* < 0.001) the glutamine effect was more pronounced in males, a 40-day extension in males compared to a 21-day extension in females (Fig. 5b).

**Fig. 6:**
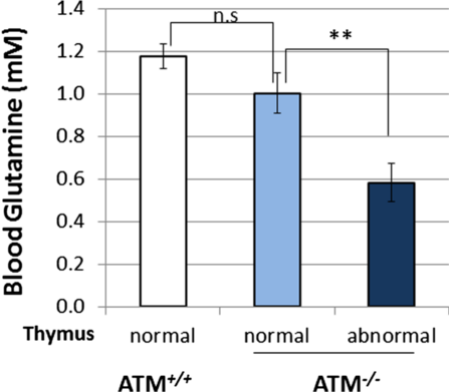
*Atm* ^*−/−*^ mice with abnormal thymus (enlarged thymus or visible thymic tumor) have significant lower blood glutamine concentration compared to *Atm* ^*−/−*^ mice with normal thymus. Wild type mice with normal thymus (WT, *n* = 15, 7 males and 8 females); *Atm−/−* mice were divided in to two groups: normal thymus (*n* = 8, 4 males and 4 females) and abnormal thymus (*n* = 6, 4 males and 2 females). Statistical significance was calculated using two-tailed *t*-test: ** = *p* < 0.01; n.s. (not significant, *p* > 0.05). Values represent means and standard errors

*Atm*^*−/−*^ mice consistently develop thymic lymphoma early in life, and almost all *Atm*^*−/−*^ mice die of these cancers [24]. During autopsy of the mice in the blood glutamine study, we noted variability in the extent of the visible thymic pathology. Recall that there is also a large variation of blood glutamine concentrations among *Atm*^*−/−*^ mice (Fig. 1). This led us to ask whether the two phenotypes might be correlated. Indeed, we found that *Atm*^*−/−*^ mice that presented with visible abnormalities in the thymus at sacrifice (apparently enlarged thymus or visible tumor) had significantly lower blood glutamine (0.6 ± 0.09 mM) than *Atm*^*−/−*^ mice with normal thymus (1.0 ± 0.09 mM – *p* < 0.01) (Fig. 6). Thus, while there is a generalized reduction of blood glutamine levels in *Atm*^*−/−*^ animals (Fig. 6), in the subset of mutants with more normal levels of glutamine, thymus pathology is significantly less likely than in those with low blood glutamine levels. As would be predicted from this observation, none of the five 10-week old *Atm*^*−/−*^ mice fed with glutamine for 2 weeks presented with abnormal thymus while half of the *Atm*^*−/−*^ littermates on regular drinking water had an abnormally enlarged thymus (Fig. 1, AWB/Glutamine blue symbol vs AWB/Control green symbol). Taken together, these observations suggest that glutamine may prolong the lifespan of *Atm*^*−/−*^ mice by slowing tumorigenesis and/or tumor progression.

### Gene expression changes in glutamine metabolism in A-T brains

**Fig. 7:**
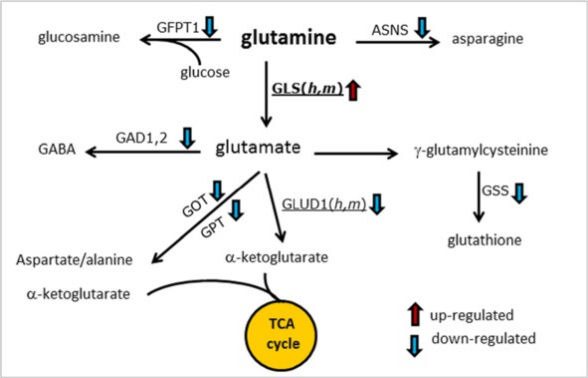
Gene expression analysis of control and ATM-deficient human and mouse brains revealed that ATM deficiency had a huge impact on genes known to take part in brain glutamine metabolism. Presented are a list of genes whose expressions were found to be significantly reduced or increased in ATM-deficient human or mouse brains (>1.5 fold change when compared to control brains, *p* < 0.05). Upregulated genes are indicated by an black upward arrow next to the gene name; downregulated genes are shown by a downward arrow. Genes whose direction of change was significant in both mouse and human data are shown with underlines

The data thus far show that glutamine supplementation is beneficial in many ways to *Atm*^*−/−*^ mice. To determine whether this finding applied to human individuals with A-T, we re-analyzed data from our previous gene expression array studies of tissue from wild type and *Atm*^*−/−*^ mouse cerebellum, as well as tissue from cerebellar cortex of control and A-T patients [17]. Given the dramatic metabolic changes brought on by ATM deficiency in mice, we queried this data set to determine if the systemic changes in the metabolomics profile were matched in the patterns of gene transcription found in brain. We focused on genes involved in glutamine metabolism whose expression was significantly altered in ATM deficiency [≥1.5 fold increase or decrease, *p* ≤ 0.05]. We found, both in human A-T and *Atm*^*−/−*^ mice, that the expression of glutaminase (GLS) was increased while the next enzyme in the pathway towards the TCA cycle, glutamate dehydrogenase (GLUD), was decreased (Fig. 7). We also found significant downregulation of several genes involved in glutamine metabolic pathways – asparagine synthetase (ASNS, glutamine hydrolyzing), glutamate decarboxylase (GAD), glutathione synthetase (GSS) and glutamine-fructose-6-phospate-transaminase (GFPT) and glutamic oxaloacetic (pyruvate) transaminase (GOT/GPT) – but these changes were only significant in the human data set. Upregulation of GLS suggests that ATM-deficient cells rely more on glutamine for energy production and is consistent with the observed reduction in serum glutamine in *Atm*^*−/−*^ animals. Down regulation of GLUD, GAD, ASNS, GSS, GFPT and GOT/GPTA in A-T brains implies a less efficient production of other molecules important for normal neuronal functions including GABA, asparagine, glucosamine, and glutathione. This may be a contributing factor in pathogenesis of A-T.

Having identified significant changes in individual gene expression, we then re-queried the entire database using Ingenuity Pathway Analysis software to help identify ensembles of genes whose expression was significantly changed in A-T brains (≥1.5 fold, *p* ≤ 0.05). Upstream Regulator Analysis (URA) identifies molecules upstream of the genes in the data set that potentially explain the observed expression changes [25]. Based on the list of significantly down regulated genes, URA predicted glutamine to be a likely upstream regulator with an overlap *p* value of 0.001 (Fisher’s exact test) and an activation Z score of 0.03. This suggests that glutamine target genes are significantly enriched in the group of genes that are down regulated in A-T. One additional finding of this analysis that we found particularly reassuring given our earlier work [4, 5] was that NFkB and TNF were predicted to be the upstream regulators for the significantly up-regulated genes, as previous studies have documented constitutional activation of NFkB signaling in ATM-deficient cells [26, 27].

### Glutamine-deprivation leads to reduced ATM expression and deregulation of the mTOR pathway

**Fig. 8:**
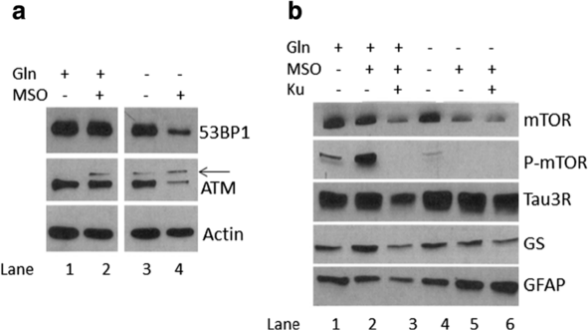
Glutamine deprivation leads to significant reduction in ATM and mTOR concentrations in neurons. **a** Both endogenous and exogenous glutamine are required for stable concentrations of ATM and 53BP1 in neurons. **b** Glutamine and ATM interact to modulate mTOR concentration and mTOR activity. The symbols above the lanes indicate the presence (+) or absence (−) of 2 mM Glutamax (gln), 5 mM methionine sulfoximine (mso), a glutamine synthase inhibitor or 10 μM KU55993 (Ku), an ATM inhibitor

To better understand the interplay between glutamine metabolism and ATM deficiency, we turned to cell culture to isolate the individual effects. We have shown that the concentrations of stress response proteins such as ATM are reduced when embryonic cortical neurons are grown in glutamine-free medium [1]. However, glutamine can be synthesized by astrocytes via glutamine synthetase (GS). Therefore, we added the GS inhibitor methionine sulfoximine (MSO) to block endogenous glutamine production (Fig. 8). Removal of either exogenous glutamine (lane 3) or endogenous glutamine with MSO (lane 2) had little or no effect by themselves. When we removed glutamine in the medium and added MSO (depletion of both exogenous and endogenous glutamine), we observed a dramatic reduction in the levels of ATM protein (lane 4). Reduced glutamine level also caused formation of oxidized ATM dimer (black arrow). Thus, the maintenance of a healthy concentration of ATM expression depends on an adequate glutamine supply. The results with a second stress response protein, 53BP1, were similar. The fact that neither of these proteins decreases in the presence of MSO alone means that an adequate supply of exogenous glutamine can overcome the loss of endogenous glutamine production. This finding validates a benefit from dietary intervention as a means of manipulating cellular glutamine concentrations. Endogenous glutamine is known to directly affect mTOR activity in multiple cell types [28]. Further, mTOR has an important reciprocal regulatory relationship with ATM. mTOR can repress ATM expression [29] while ROS-induced ATM activation can repress mTOR activity [30]. Together, these observations suggest the possibility that the effects of glutamine on ATM expression may be mediated by the mTOR pathway.

In cultured wild-type mouse neurons, low exogenous glutamine almost completely suppresses mTOR phosphorylation (Fig. 8b, compare lane 4 to lane 1), while inhibiting endogenous glutamine production increases it (compare lane 2 to lane 1). This increase is completely dependent on exogenous glutamine (compare lane 5 to lane 2), as well as ATM activity (compare lane 2 to lane 3). These observations confirm the relation between glutamine, ATM and mTOR. Of note is the fact that the basic cytoarchitecture of the cells, as measured by the concentrations of the microtubule associated protein tau, seems largely unaffected by either ATM or GS inhibition.

### ATM-deficient neurons are more dependent on exogenous glutamine

ATM is required throughout the lifetime of a neuron. It is the primary mediator of the response to DNA double strand breaks that arise from exposure to ionizing radiation. However, cells lacking ATM are also hypersensitive to insults other than double strand breaks, particularly oxidative stress. Since glutamine is essential for these responses [1], we hypothesized that ATM-deficient cells in culture would be more dependent on exogenous glutamine.

**Fig. 9:**
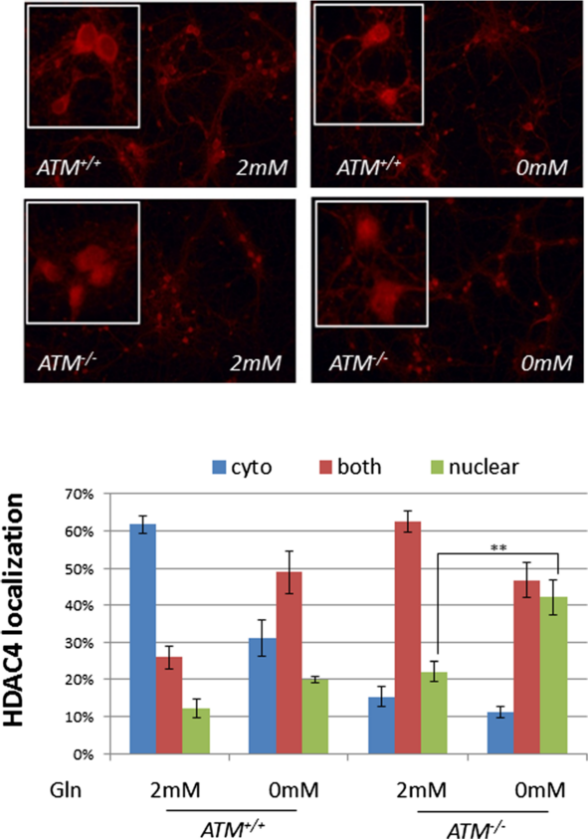
*Atm* ^−/−^ neurons are significantly more sensitive to glutamine deficiency. Wild type and *Atm* ^−/−^ primary neurons were grown in culture media with or without glutamine supplementation for 24 h, and then stained with HDAC4. For quantification, 5 random fields of 200 cells for each condition were counted

Recent studies from our lab have shown that ATM regulates the cytoplasmic location of histone deacetylase 4 (HDAC4) [31]. Because ATM deficiency causes nuclear accumulation of HDAC4 in neurons and promotes neurodegeneration, we evaluated cultured *Atm*^*−/−*^ and wild-type neurons’ response to glutamine deficiency by monitoring HDAC4 localization (Fig. 9). We found that low glutamine caused a significant increase in the percentage of neurons with nuclear HDAC4 in both genotypes (nuclear HDAC4, green bars). However, even when given glutamine, the localization of HDAC4 in *Atm*^*−/−*^ neurons was still far from normal. Only 10 % of the *Atm*^*−/−*^ neurons maintained an exclusively cytoplasmic localization of HDAC4 upon removal of glutamine compared to over 60 % in the wild type (Fig. 9, blue bars). Notably, *Atm*^*−/−*^ neurons grown in low glutamine have significantly higher percentage of cells contained nuclear HDAC4; about two fold increase (Fig. 9, green bars). These data suggest that ATM-deficient neurons are heavily dependent on exogenous glutamine supplementation to maintain their normal epigenetic landscape.

**Fig. 10:**
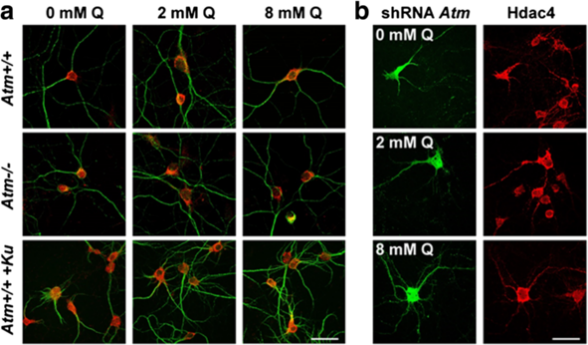
The effect of glutamine on HDAC4 localization. **a** DIV14 neurons from Atm wildtype (*Atm* ^*+/+*^), or knockout (*Atm* ^*−/−*^) were cultured under various dosages of glutamine for 72 h. To access the role of ATM kinase activity, treatment with 2 μM of ATM specific inhibitor (KU-60019) was initiated in DIV9 wildtype neurons for 48 h in complete medium, followed by glutamine treatment at different dosage for another 72 h. **b** shRNA against Atm was also used to study the effect of losing the protein in mature neurons. shRNA was transfected into DIV9 wildtype neurons for 48 h in complete medium, followed by glutamine treatment at different dosage for another 72 h. Effect on HDAC4 localization was analyzed by immunohistochemistry. Scale Bar = 25 μm. *N* = 3

Considering the similarities in nuclear HDAC4 populations between glutamine-deprived wild-type neurons and *Atm*^*−/−*^ neurons in normal media, we hypothesized that increased glutamine supplementation would be beneficial to ATM-deficient cells. To test this idea, we performed an extensive analysis on the effect of glutamine dosages (0, 2 and 8 mM) on HDAC4 localization. In DIV14 wildtype *Atm*^*+/+*^ neurons, elimination of glutamine in the culture led to modest HDAC4 nuclear localization. The presence of glutamine in the culture medium completely prevented the nuclear presence of HDAC4 (Fig. 10). In contrast, in models of ATM deficiencies such as neurons isolated from *Atm*^*−/−*^ mice, wildtype neurons treated with ATM specific inhibitor Ku-66019 and wildtype neurons with endogenous *Atm* knocked down with shRNA, removing glutamine from the culture medium robustly rendered HDAC4 to be localized in the nucleus. The presence of glutamine prevented such phenomena, and the effect became more prominent when glutamine supplementation was high (8 mM). In summary, glutamine reversed HDAC4 nuclear translocation (epigenetic changes) resulting from ATM-deficiency whether due to genetic deficiency (*Atm*^*−/−*^), acute knockdown (shRNA), or the pharmacological inhibition of enzyme activity of ATM (Ku).

**Fig. 11:**
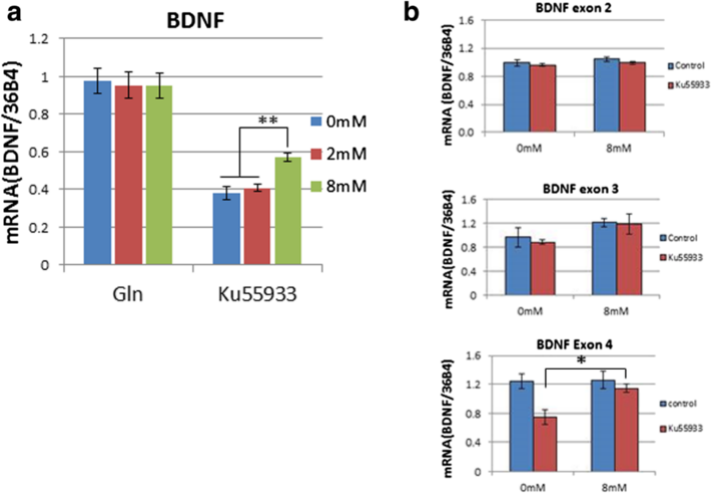
Supramolar glutamine supplementation partially rescued ATM activity loss induced reduction of BDNF expression. Wild type primary neurons DIV14 were grown in medium containing 0 mM, 2 mM or 8 mM glutamine for 3 days and collected for gene expression analysis. **a** Quantification of the BDNF common exon by real-time PCR. **b** BDNF exon 2, 3 and 4 expression levels measured by RT-PCR

To measure the glutamine effect with a more quantitative method, we turned to brain-derived neurotrophic factor (Bdnf) gene expression. Our previous studies have shown that Bdnf expression is significantly down regulated in ATM-deficient neurons [31]. Late phase LTP is known to be dependent on BDNF [32] and, as our physiological experiments have shown, the effect of glutamine is most prominent at these times suggesting a linkage (Fig. 4a). To test this idea, cultured wild type neurons were treated with KU59933 and grown in 0, 2 and 8 mM glutamine. As shown in Fig. 11, neurons grown in 8 mM glutamine expressed significantly higher levels of BDNF message (the common exon) than those grown in 0–2 mM glutamine (Fig. 11a). Transcription of the mouse Bdnf gene is controlled by more than 9 promoters, resulting in at least 18 transcripts. Interestingly, individual splice variants of the BDNF message responded differently to glutamine. 8 mM glutamine increased the expression of exon4 significantly in Ku treated neurons but had no effect on untreated neurons (Fig. 11b). Given that exon 4 is critical for GABAergic transmission and plasticity of frontal cortex [33], these observations suggest that glutamine may act on LTP through restoration of the concentrations of Bdnf in ATM-deficient neurons.

## Discussion

Cultured neurons exposed to exogenous stressors such as DNA damage, heavy metals, oxidation or the Aβ peptide have heightened vulnerability to glutamine deprivation [1]. In mouse models of familial Alzheimer’s disease (AD), oral glutamine supplementation reduces phosphorylated tau as well as the ectopic appearance of neuronal cell cycle proteins [1]. We have now extended these AD-related studies to a second neurodegenerative disease, ataxia-telangiectasia (A-T). The AD model we tested was the R1.40 mouse [34]. These animals are a faithful genetic mimic of early-onset APP-driven dominantly inherited forms of human familial AD. Ataxia-telangiectasia is a recessive genetic condition of childhood neurodegenerative disease caused by mutations in the ATM gene. It is a true multi-systemic disease, but the neurological symptoms are the most prevalent and the most debilitating. On the surface, AD and A-T seem like very differences diseases, but our research shows that glutamine has a positive impact on the phenotype of both. This may be mediated through ATM, as we have recently discovered that AD patients often exhibit ATM deficiency, as seen through HDAC4 nuclear translocation in hippocampal neurons [35]. Our finding on the powerful neuroprotective effects of glutamine in mouse models in both AD and A-T suggests that its benefits are not disease specific, and may be extrapolated to other neurodegenerative disorders – even beyond AD and A-T. Indeed, Rozas et al. [36] have recently shown that glutamine supplementation prolongs the survival of mice with tuberous sclerosis, an autosomal dominant neurodevelopmental disease.

Our discovery that glutamine and ATM interact to influence blood glucose, body weight and the phosphorylation of mTOR emphasizes that, beyond the DNA damage response, ATM has deep connections with the fundamental pathways of cellular metabolism. This largely cytoplasmic function of ATM is increasingly recognized [30, 37–39] and applies to energy metabolism in mitochondria as well. Indeed, A-T patients have an unusual form of diabetes [21], and present with irregularities in their glucose utilization [40]. A recent study also revealed that adult asymptomatic relatives of A-T patients (who would be expected to carry a single mutated allele of ATM) have decreased glucose metabolism in cerebellar vermis and hippocampus [22]. Additionally, we note that loss of retinoblastoma (pRB) results in reduced glucose utilization and enhanced susceptibility to oxidative stress. Glutamine rescues these effects by providing an alternative to glucose oxidation and enhancing glutathione production [41]. The suggestion is that the effects of glutamine on ATM-deficient neurons that we report may share similarities with RB-deficient cells. The findings by Rozas et. al., showing that glutamine supplementation could prolong survival of mice with tuberous sclerosis, take on added significance in this context. Tsc2 knockout mice supplemented with glutamine survive 2 weeks longer than those without glutamine supplement, a 20 % increase in lifespan [36]. TSC2 is a potent mTOR suppressor and it is noteworthy that ATM activates TSC2 to repress mTOR complex1 in response to oxidative stress [30]. These observations support our suggestion that glutamine may exert its protective effect on *Atm*^*−/−*^ mice by modulating mTOR activity. It is likely that part of this pathway passes through an ATM node, but the finding of Rozas suggest that passage through a TSC2 node is also possible.

Glutamine is not itself a neurotransmitter but it serves as an immediate precursor for glutamate and is only two steps removed from GABA. It has been shown that presynaptic glutamine transport is involved in the maintenance of excitatory synaptic currents [42], and glutamine supplementation rescues the rapid synaptic GABA depletion induced by astrogliosis, thus restoring inhibitory synaptic currents in mouse CA1 neurons [25]. Given that ATM is involved in neuronal vesicle trafficking [16] and its concentrations are sensitive to the availability of glutamine (Fig. 8 and reference [1]) it is tempting to speculate that glutamine increases the small amounts of residual ATM protein found in the *Atm*^*−/−*^ brain [39] thus partially restoring function.

Several additional features of the LTP experiments deserve mention. First, because our measurements were made in isolated brain slices over a period of several hours, it would appear that the effects of glutamine are long-term in nature rather than reflecting short-term, moment-to-moment, changes in transmitters. The recording medium, in which the slices were bathed during our measurements, contained only balanced salt solution and no added glutamine. Therefore, the LTP differences must represent chronic changes that remain stable even after the extracellular environment of the synapse is changed. Second, RT-PCR results revealed a partial rescue of BDNF mRNA (common exon) and almost total restoration of exon 4 expression after glutamine supplementation (8 mM) in KU-treated neurons (Fig. 11). This, combined with the fact that glutamine’s effects on LTP are strongest in late-LTP, which is dependent on BDNF [32], makes it possible that glutamine acts through restoration of BDNF in the same way that it improves stress-response proteins such as 53BP1, ATM, ATR, etc.

Untreated *Atm*^*−/−*^ mice present with lower blood glutamine concentrations suggesting that glutamine metabolism may be impaired in A-T patients as well. The expression data summarized in Fig. 7 are consistent with this conclusion, and the independent URA analysis further supports the importance of glutamine in the changes in homeostasis brought on by ATM deficiency. Examination of the pathways involved suggests that while glutamine conversion to glutamate is likely upregulated in A-T, the pathways leading beyond glutamate are reduced (at least in brain). This would be predicted to deplete local glutamine stores, possibly at the expense of side pathways leading to asparagine and glucosamine (Fig. 7). This decrease in side pathway products could have a multitude of negative effects. For example, decreased asparagine would be expected to be destructive. ASNS-deficiency leads to progressive cerebral atrophy and intellectual disability [43] and can cause severe neurological impairment without involvement of peripheral tissues. Because of the poor transport of asparagine across the blood–brain barrier, the brain depends on local synthesis, suggesting that even a small block in its synthesis could have huge effects on brain function [44]. Glutamine is also an important precursor for de novo synthesis of arginine in humans [45]. Recent studies suggest that low level of total brain arginine contribute to neurodegeneration in Alzheimer’s disease [46]; a similar pathway may work in A-T brains too. Decreased GFPT expression predicts a lower glucosamine production in A-T brains. Glucosamine exerts a neuroprotective effect via suppression of inflammation through its ability to inhibit NFkB activation [47]; therefore, lower glucosamine would forecast higher NFkB activity in A-T brains. Other changes predicted from the expression array data enhance the picture further. For example, α-ketoglutarate is a major metabolite that feeds directly into TCA cycle and its reduction would result in lower cellular energy. Reduced GAD can lead to reduced GABA synthesis and decreased GABAergic transmission. Lastly, since GSS catalyzes the final step of glutathione production and its expression was significantly reduced in A-T, a situation of lower glutathione production with concomitant reduction in anti-oxidant defenses would be predicted.

Glutamine is known to support fast growing cells in culture and in tumor grafts [48]. Indeed tumor cells are often described as ‘addicted’ to glutamine [49]. An oft-cited reason for the low life expectancy of *Atm*^*−/−*^ mice (about 3 months in our colony) is their high cancer predilection. Almost all *Atm*^*−/−*^ mice die of thymic lymphomas. Thus we initially worried that, despite its significant neuroprotective properties, by elevating systemic concentrations of glutamine to improve nervous function we would be promoting the establishment and growth of tumors in the *Atm*^*−/−*^ mice. Rather than causing premature death, however, oral glutamine supplementation paradoxically increased the lifespan of the *Atm*^*−/−*^ mice by about one-third. In fact, *Atm*^*−/−*^ mice supplemented with glutamine appeared to have slower tumor progression (Fig. 6). In line with our observations in *Atm*^*−/−*^ mice, it has been shown that rats with carcinoma had significantly lower muscle glutamine content and the size of tumor negatively correlated with the glutamine level [50]. Significantly lower plasma glutamine level has also been reported in breast cancer patients and in male gastrointestinal cancer patients [51]. The implication is that glutamine’s effect on tumor growth is context-dependent [52]. Both human and animal studies suggest that glutamine can be given to breast cancer patients without stimulating tumor growth or metastasis. The reasons for this are unknown, but glutamine generally strengthens the immune system, especially the natural killer cells, and thus might boost the body’s own defenses against cancer [53–55]. Whatever the ultimate explanation, it would appear in the right clinical circumstances it is possible to take advantage of the ability of glutamine to improve brain function yet not hasten death due to cancer.

## Conclusions

We have shown previously that glutamine is neuroprotective in vitro and in mouse models of AD. We have now extended these AD-related studies to A-T. Unlike AD, A-T is entirely a genetic disease, yet the epigenetic landscape of the chromatin is part of the realization of the phenotype. To the extent that the disease symptoms result in part from these epigenetic changes, it is reasonable to predict that environmental factors can alter the timing and perhaps the extent of various disease events. Our data suggest that glutamine is a powerful part of an organism’s internal environment, and that changes in its concentrations have a huge impact on the function of the organism in general and the brain in particular. Glutamine supplementation is a promising therapeutic candidate for the treatment of human AD, A-T and beyond.

## Abbreviations

53BP1, p53-binding protein 1; AD, Alzheimer’s disease; A-T, ataxia telangiectasia; ATM, ataxia telangiectasia mutated; BDNF, brain-derived neurotrophic factor; CA1, cornus ammonis 1, region 1 of hippocampus proper; fEPSPs, extracellular recordings of field excitatory postsynaptic potential; GABA, γ-aminobutyric acid; GAD, glutamate decarboxylase; GEO, gene expression omnibus; Gln or Q, glutamine; GLS, glutaminase; GOT/GPT, glutamic oxaloacetic (pyruvate) transaminase; GSS, glutathione synthetase; HDAC4, histone deacetylase 4; IL8, interleukin 8; LTP, Long term potentiation; MSO, methionine sulfoximine; mTOR, mammalian target of rapamycin; ROS, reactive oxygen species; RT-PCR - reverse transcription polymerase chain reaction; shRNA - short hairpin RNA; TBS, theta burst stimulation; TCA, tricarboxylic acid cycle; TSC2, Tuberous Sclerosis Complex 2
