# Erratum to: The impact of glutamine supplementation on the symptoms of ataxia-telangiectasia: a preclinical assessment

**DOI:** 10.1186/s13024-017-0151-6

**Published:** 2017-01-12

**Authors:** Jianmin Chen, Yanping Chen, Graham Vail, Heiman Chow, Yang Zhang, Lauren Louie, Jiali Li, Ronald P. Hart, Mark R. Plummer, Karl Herrup

**Affiliations:** 1 Department of Cell Biology and Neuroscience, Rutgers University, 604 Allison Road, Piscataway, NJ 08854 USA; 2 Division of Life Science, The Hong Kong University of Science and Technology, Clear Water Bay, Kowloon, Hong Kong; 3 Kunming Institute of Zoology, Chinese Academy of Sciences, Kunming, Yunnan China

## Erratum

The original article [1] mistakenly omitted mention of a grant support source in the Funding sub-section, and would like to acknowledge that the work was also supported by National Key Basic Research Program of China (2013CB530900).
